# Supplementary material for: Trace Elements in Beef Cattle: A Review of the Scientific Approach from One Health Perspective
Source: Animals (Basel). 2022 Aug 31;12(17):2254. doi: 10.3390/ani12172254 (PMC9454500; doi:10.3390/ani12172254)
Supplement: Supplementary file 1 [file animals-12-02254-s001.zip › animals-1843585-supplementary File S1.pdf]

SUPPLEMENTARY FILE-S1

Table S1. Search keywords and strings used to retrieve papers regarding trace elements in beef cattle production published in the period 2000-2022, from Scopus, Web of Science and PubMed databases.

**Sets**	**Keywords**	**Strings of Search**			
S1	(trace OR element OR metal)	**In Title**		**In Abstract**	
S2	(meat OR beef OR tissue OR muscle OR liver OR kidney OR blood OR hair OR plasma OR serum)	(S5)			AND
		[(S1 AND S2)	AND	(S3 AND S1 AND S2)	OR
S3	(cattle OR livestock OR bovine OR farm OR animal OR slaughter* OR food OR steer OR heifer OR calf)	(S1 AND S2)	AND	(S4 AND S1 AND S2)	OR
		(S1 AND S3)	AND	(S2 AND S1 AND S3)	OR
		(S1 AND S3)	AND	(S4 AND S1 AND S3)	OR
S4	(zinc OR selenium OR copper OR chromium OR iodine OR molybdenum OR manganese OR silicon OR nickel OR boron OR vanadium OR arsenic OR cadmium OR lead OR mercury OR aluminum OR fluorine OR lithium OR tin OR cobalt)	(S1 AND S4)	AND	(S2 AND S1 AND S4)	OR
		(S1 AND S4)	AND	(S3 AND S1 AND S4)	OR
		(S2 AND S3)	AND	(S1 AND S2 AND S3)	OR
		(S2 AND S3)	AND	(S4 AND S2 AND S3)	OR
		(S2 AND S4)	AND	(S1 AND S2 AND S4)	OR
		(S2 AND S4)	AND	(S3 AND S2 AND S4)	OR
S5	(cattle OR livestock OR bovine OR beef OR steer OR pasture OR heifer OR calf)	(S3 AND S4)	AND	(S1 AND S3 AND S4)	OR
		(S3 AND S4)	AND	(S2 AND S3 AND S4)]	NOT[a]

[a] The Boolean operator NOT was used with words in abstracts such as “milk”, “albumin” and “genome” to restrict results of the search.

Sentinel papers used:

1. Chibunda, R.T.; Janssen, C.R. Mercury Residues in Free-Grazing Cattle and Domestic Fowl Form the Artisanal Gold Mining Area of Geita District, Tanzania. Food Addit. {\&} Contam. Part A 2009, 26, 1482–1487, doi:10.1080/02652030903114928.
2. Rudy, M. Correlation of Lead, Cadmium and Mercury Levels in Tissue and Liver Samples with Age in Cattle. Food Addit. {\&} Contam. Part A 2009, 26, 847–853, doi:10.1080/02652030902835747.
3. van der Fels-Klerx, I.; Römkens, P.; Franz, E.; van Raamsdonk, L. Modeling Cadmium in the Feed Chain and Cattle Organs. Biotechnol. Agron. Soc. Env. 2011, 15, 53–59.
4. Khalafalla, F.A.; Ali, F.H.; Schwagele, F.; Abd-El-Wahab, M.A. Heavy Metal Residues in Beef Carcasses in Beni-Suef Abattoir, Egypt. Vet. Italliana 2011, 47, 351–361.
5. Adetunji, V.O.; Famakin, I.O.; Chen, J. Lead and Cadmium Levels in Cattle Muscle and Edible Tissues Collected from a Slaughter Slab in Nigeria. Food Addit. Contam. Part B 2014, 7, 79–83, doi:10.1080/19393210.2013.848942.
6. Alkmim Filho, J.F.; Germano, A.; Dibai, W.L.S.; Vargas, E.A.; Melo, M.M. Heavy Metals Investigation in Bovine Tissues in Brazil. Food Sci. Technol. 2014, 34, 110–115, doi:10.1590/S0101-20612014005000013.
7. Canty, M.J.; Scanlon, A.; Collins, D.M.; McGrath, G.; Clegg, T.A.; Lane, E.; Sheridan, M.K.; More, S.J. Cadmium and Other Heavy Metal Concentrations in Bovine Kidneys in the Republic of Ireland. Sci. Total Environ. 2014, 485–486, 223–231, doi:10.1016/j.scitotenv.2014.03.065.
8. Sakakibara, M.; Sera, K.; Kurniawan, I.A. Mercury Contamination of Cattle in Artisanal and Small-Scale Gold Mining in Bombana, Southeast Sulawesi, Indonesia. Geosciences 2017, 7, 133, doi:10.3390/geosciences7040133.
9. Kasozi, K.I.; Natabo, P.C.; Namubiru, S.; Tayebwa, D.S.; Tamale, A.; Bamaiyi, P.H. Food Safety Analysis of Milk and Beef in Southwestern Uganda. J. Environ. Public Health 2018, 2018, doi:10.1155/2018/1627180.
10. Hashemi, M. Heavy Metal Concentrations in Bovine Tissues (Muscle, Liver and Kidney) and Their Relationship with Heavy Metal Contents in Consumed Feed. Ecotoxicol. Environ. Saf. 2018, 154, 263–267, doi:10.1016/j.ecoenv.2018.02.058.
11. de Souza Ramos, B.; Pestana, I.A.; Caldas, D.; Azevedo, L.S.; Almeida, M.G.; de Souza, C.M.M. Exposure to Toxic and Essential Trace Elements through the Intake of Processed. Environ. Monit. Assess. 2019, 191, 477, doi:10.1007/s10661-019-7618-6.
12. Rodríguez-Marín, N.; Hardisson, A.; Gutiérrez, Á.J.; Luis-González, G.; González-Weller, D.; Rubio, C.; Paz, S. Toxic (Al, Cd, and Pb) and Trace Metal (B, Ba, Cu, Fe, Mn, Sr, and Zn) Levels in Tissues of Slaughtered Steers: Risk Assessment for the Consumers. Environ. Sci. Pollut. Res. 2019, 26, 28787–28795, doi:10.1007/s11356-019-06090-1.
13. Skalny, A. V; Salnikova, E. V; Burtseva, T.I.; Skalnaya, M.G.; Tinkov, A.A. Zinc, Copper, Cadmium, and Lead Levels in Cattle Tissues in Relation to Different Metal Levels in Ground Water and Soil. Environ. Sci. Pollut. Res. 2019, 26, 559–569, doi:10.1007/s11356-018-3654-y.
14. Khan, Z.I.; Akhtar, M.; Ahmad, K.; Ashfaq, A.; Nadeem, M.; Bashir, H.; Munir, M.; Malik, I.S. A Study on the Seasonal Transfer of Two Metals from Pasture to Animals: Health Risk Assessment. Environ. Sci. Pollut. Res. 2020, 27, 16339–16349, doi:10.1007/s11356-020-08140-5.
15. Nawrocka, A.; Durkalec, M.; Szkoda, J.; Filipek, A.; Kmiecik, M.; Żmudzki, J.; Posyniak, A. Total Mercury Levels in the Muscle and Liver of Livestock and Game Animals in Poland, 2009–2018. Chemosphere 2020, 258, 127311, doi:10.1016/j.chemosphere.2020.127311.

BELOW IS A SEARCH STRING ADAPTED FOR SCOPUS' ADVANCED SEARCH BOX:

((TITLE(cattle OR livestock OR bovine OR beef OR steer OR pasture OR heifer OR calf)))

AND (((TITLE((trace OR element OR metal) AND (meat OR beef OR tissue OR muscle OR liver OR kidney OR blood OR hair OR plasma OR serum))) AND (ABS((cattle OR livestock OR bovine OR farm OR animal OR slaughter OR slaughtered OR slaughtered OR food OR steer OR heifer OR calf) AND (trace OR element OR metal)AND (meat OR beef OR tissue OR muscle OR liver OR kidney OR blood OR hair OR plasma OR serum))))

OR ((TITLE((trace OR element OR metal)   AND (meat OR beef OR tissue OR muscle OR liver OR kidney OR blood OR hair OR plasma OR serum))) AND (ABS((arsenic OR cadmium OR lead OR mercury OR Aluminum OR Fluorine OR lithium OR stannum OR tin OR zinc OR selenium OR copper OR chromium OR iodine OR molybdenum OR manganese OR silicon OR nickel OR boron OR vanadium OR cobalt)   AND (trace OR element OR metal) AND (meat OR beef OR tissue OR muscle OR liver OR kidney OR blood OR hair OR plasma OR serum))))

OR ((TITLE((trace OR element OR metal) AND (cattle OR livestock OR bovine OR farm OR animal OR slaughter OR slaughtered OR food OR steer OR heifer OR calf))) AND (ABS((meat OR beef OR tissue OR muscle OR liver OR kidney OR blood OR hair OR plasma OR serum) AND (trace OR element OR metal) AND (cattle OR livestock OR bovine OR farm OR animal OR slaughter OR slaughtered OR food OR steer OR heifer OR calf))))

OR ((TITLE((trace OR element OR metal) AND (cattle OR livestock OR bovine OR farm OR animal OR slaughter OR slaughtered OR food OR steer OR heifer OR calf))) AND (ABS((arsenic OR cadmium OR lead OR mercury OR Aluminum OR Fluorine OR lithium OR stannum OR tin OR zinc OR selenium OR copper OR chromium OR iodine OR molybdenum OR manganese OR silicon OR nickel OR boron OR vanadium OR cobalt) AND (trace OR element OR metal) AND (cattle OR livestock OR bovine OR farm OR animal OR slaughter OR slaughtered OR food OR steer OR heifer OR calf))))

OR ((TITLE((trace OR element OR metal) AND (arsenic OR cadmium OR lead OR mercury OR Aluminum OR Fluorine OR lithium OR stannum OR tin OR zinc OR selenium OR copper OR chromium OR iodine OR molybdenum OR manganese OR silicon OR nickel OR boron OR vanadium OR cobalt))) AND (ABS((meat OR beef OR tissue OR muscle OR liver OR kidney OR blood OR hair OR plasma OR serum) AND (trace OR element OR metal) AND (arsenic OR cadmium OR lead OR mercury OR Aluminum OR Fluorine OR lithium OR stannum OR tin OR zinc OR selenium OR copper OR chromium OR iodine OR molybdenum OR manganese OR silicon OR nickel OR boron OR vanadium OR cobalt))))

OR ((TITLE((trace OR element OR metal) AND (arsenic OR cadmium OR lead OR mercury OR Aluminum OR Fluorine OR lithium OR stannum OR tin OR zinc OR selenium OR copper OR chromium OR iodine OR molybdenum OR manganese OR silicon OR nickel OR boron OR vanadium OR cobalt))) AND (ABS((cattle OR livestock OR bovine OR farm OR animal OR slaughter OR slaughtered OR food OR steer OR heifer OR calf) AND (trace OR element OR metal) AND (arsenic OR cadmium OR lead OR mercury OR Aluminum OR Fluorine OR lithium OR stannum OR tin OR zinc OR selenium OR copper OR chromium OR iodine OR molybdenum OR manganese OR silicon OR nickel OR boron OR vanadium OR cobalt))))

OR ((TITLE((meat OR beef OR tissue OR muscle OR liver OR kidney OR blood OR hair OR plasma OR serum) AND (cattle OR livestock OR bovine OR farm OR animal OR slaughter OR slaughtered OR food OR steer OR heifer OR calf))) AND (ABS((trace OR element OR metal) AND (meat OR beef OR tissue OR muscle OR liver OR kidney OR blood OR hair OR plasma OR serum) AND (cattle OR livestock OR bovine OR farm OR animal OR slaughter OR slaughtered OR food OR steer OR heifer OR calf))))

OR ((TITLE((meat OR beef OR tissue OR muscle OR liver OR kidney OR blood OR hair OR plasma OR serum) AND (cattle OR livestock OR bovine OR farm OR animal OR slaughter OR slaughtered OR food OR steer OR heifer OR calf))) AND (ABS((arsenic OR cadmium OR lead OR mercury OR Aluminum OR Fluorine OR lithium OR stannum OR tin OR zinc OR selenium OR copper OR chromium OR iodine OR molybdenum OR manganese OR silicon OR nickel OR boron OR vanadium OR cobalt) AND (meat OR beef OR tissue OR muscle OR liver OR kidney OR blood OR hair OR plasma OR serum) AND (cattle OR livestock OR bovine OR farm OR animal OR slaughter OR slaughtered OR food OR steer OR heifer OR calf))))

OR ((TITLE((meat OR beef OR tissue OR muscle OR liver OR kidney OR blood OR hair OR plasma OR serum) AND (arsenic OR cadmium OR lead OR mercury OR Aluminum OR Fluorine OR lithium OR stannum OR tin OR zinc OR selenium OR copper OR chromium OR iodine OR molybdenum OR manganese OR silicon OR nickel OR boron OR vanadium OR cobalt))) AND (ABS((trace OR element OR metal) AND (meat OR beef OR tissue OR muscle OR liver OR kidney OR blood OR hair OR plasma OR serum) AND (arsenic OR cadmium OR lead OR mercury OR Aluminum OR Fluorine OR lithium OR stannum OR tin OR zinc OR selenium OR copper OR chromium OR iodine OR molybdenum OR manganese OR silicon OR nickel OR boron OR vanadium OR cobalt))))

OR ((TITLE((meat OR beef OR tissue OR muscle OR liver OR kidney OR blood OR hair OR plasma OR serum) AND (arsenic OR cadmium OR lead OR mercury OR Aluminum OR Fluorine OR lithium OR stannum OR tin OR zinc OR selenium OR copper OR chromium OR iodine OR molybdenum OR manganese OR silicon OR nickel OR boron OR vanadium OR cobalt))) AND (ABS((cattle OR livestock OR bovine OR farm OR animal OR slaughter OR slaughtered OR food OR steer OR heifer OR calf) AND (meat OR beef OR tissue OR muscle OR liver OR kidney OR blood OR hair OR plasma OR serum) AND (arsenic OR cadmium OR lead OR mercury OR Aluminum OR Fluorine OR lithium OR stannum OR tin OR zinc OR selenium OR copper OR chromium OR iodine OR molybdenum OR manganese OR silicon OR nickel OR boron OR vanadium OR cobalt))))

OR ((TITLE((cattle OR livestock OR bovine OR farm OR animal OR slaughter OR slaughtered OR food OR steer OR heifer OR calf) AND (arsenic OR cadmium OR lead OR mercury OR Aluminum OR Fluorine OR lithium OR stannum OR tin OR zinc OR selenium OR copper OR chromium OR iodine OR molybdenum OR manganese OR silicon OR nickel OR boron OR vanadium OR cobalt))) AND (ABS((trace OR element OR metal) AND (cattle OR livestock OR bovine OR farm OR animal OR slaughter OR slaughtered OR food OR steer OR heifer OR calf) AND (arsenic OR cadmium OR lead OR mercury OR Aluminum OR Fluorine OR lithium OR stannum OR tin OR zinc OR selenium OR copper OR chromium OR iodine OR molybdenum OR manganese OR silicon OR nickel OR boron OR vanadium OR cobalt))))

OR ((TITLE((cattle OR livestock OR bovine OR farm OR animal OR slaughter OR slaughtered OR food OR steer OR heifer OR calf) AND (arsenic OR cadmium OR lead OR mercury OR Aluminum OR Fluorine OR lithium OR stannum OR tin OR zinc OR selenium OR copper OR chromium OR iodine OR molybdenum OR manganese OR silicon OR nickel OR boron OR vanadium OR cobalt))) AND (ABS((meat OR beef OR tissue OR muscle OR liver OR kidney OR blood OR hair OR plasma OR serum) AND (cattle OR livestock OR bovine OR farm OR animal OR slaughter OR slaughtered OR food OR steer OR heifer OR calf) AND (arsenic OR cadmium OR lead OR mercury OR Aluminum OR Fluorine OR lithium OR stannum OR tin OR zinc OR selenium OR copper OR chromium OR iodine OR molybdenum OR manganese OR silicon OR nickel OR boron OR vanadium OR cobalt)))))

AND NOT ((ABS(rna OR dna OR "albumin" OR "gene" OR "genes" OR "genome" OR "molecular" OR "infection" OR cell OR response OR "genetic" OR "performance" OR "milk" OR suppl)) AND NOT (ABS((performance AND (liver OR blood OR meat OR beef OR status)) OR (milk AND (beef OR*

meat OR blood OR liver)) OR(suppl AND (liver OR blood OR plasma OR status)) OR(cell AND (liver OR kidney OR meat)) OR(genetic AND (muscle)))))*

AND (LIMIT-TO (PUBYEAR,2022) OR (PUBYEAR,2021) OR (PUBYEAR,2020) OR LIMIT-TO (PUBYEAR,2019) OR LIMIT-TO (PUBYEAR,2018) OR LIMIT-TO (PUBYEAR,2017) OR LIMIT-TO (PUBYEAR,2016) OR LIMIT-TO (PUBYEAR,2015) OR LIMIT-TO (PUBYEAR,2014) OR LIMIT-TO (PUBYEAR,2013) OR LIMIT-TO (PUBYEAR,2012) OR LIMIT-TO (PUBYEAR,2011) OR LIMIT-TO (PUBYEAR,2010) OR LIMIT-TO (PUBYEAR,2009) OR LIMIT-TO (PUBYEAR,2008) OR LIMIT-TO (PUBYEAR,2007) OR LIMIT-TO (PUBYEAR,2006) OR LIMIT-TO (PUBYEAR,2005) OR LIMIT-TO (PUBYEAR,2004) OR LIMIT-TO (PUBYEAR,2003) OR LIMIT-TO (PUBYEAR,2002) OR LIMIT-TO (PUBYEAR,2001) OR LIMIT-TO (PUBYEAR,2000)) AND (LIMIT-TO (DOCTYPE,"ar"))
